# Biocontrol Ability and Action Mechanism of *Meyerozyma guilliermondii* 37 on Soft Rot Control of Postharvest Kiwifruit

**DOI:** 10.3390/microorganisms10112143

**Published:** 2022-10-29

**Authors:** Hui Pan, Caihong Zhong, Zupeng Wang, Lei Deng, Wenyi Li, Juan Zhao, Chao-an Long, Li Li

**Affiliations:** 1 Key Laboratory of Plant Germplasm Enhancement and Specialty Agriculture, Engineering Laboratory for Kiwifruit Industrial Technology, Wuhan Botanical Garden, Chinese Academy of Sciences, Wuhan 430074, China; 2 Key Laboratory of Horticultural Plant Biology of Ministry of Education, National R&D Center for Citrus Postharvest Technology, Huazhong Agricultural University, Wuhan 430070, China

**Keywords:** kiwifruit, soft rot, *Meyerozyma guilliermondii*, biocontrol

## Abstract

Postharvest soft rot of kiwifruit has resulted in substantial market losses, yet there were few antagonistic yeasts reported to control the disease. This study screened 1113 yeast strains for potential antagonistic yeast to control soft rot of kiwifruit caused by *Botryosphaeria dothidea* and *Diaporthe actinidiae*, and strain 37 was selected to evaluate the control efficacy and mechanisms, which was identified as *Meyerozyma guilliermondii* via molecular biological identification. Our results showed that *M. guilliermondii* 37 effectively reduced pathogen spore germination rate to 28.52% and decay incidence of inoculated kiwifruit to 42.11% maximumly, whereas cell-free supernatant lacked antifungal activity, implying that *M. guilliermondii* 37 didn’t produce direct antifungal compounds against the two pathogens. In addition, *M. guilliermondii* 37 adhered tenaciously to the pathogens’ mycelium and colonized rapidly in kiwifruit flesh. Moreover, yeast strain 37 induced kiwifruit resistance by elevating the defense-related enzyme activity, increasing the antioxidant substances content, and suppressing the cell wall-degrading enzyme activity. Gene expression was consistent with the corresponding enzyme activity. Further postharvest yeast immersion treatment significantly reduced natural decay to 35.69% while maintaining soft-ripe quality. These results indicated that *M. guilliermondii* 37 might serve as a biocontrol agent against postharvest soft rot in kiwifruit.

## 1. Introduction

Kiwifruit is a widely consumed fruit due to its delicate flavor and high nutrient content. According to a recent study conducted in 2021 by the Kiwifruit Section of the Chinese Society for Horticultural Science, the total annual production of kiwifruit (*Actinidia chinensis*) in our nation was 3.22 million t in 2020 [1]. However, it is susceptible to postharvest diseases during its storage, transportation, and selling periods. In particular, soft rot caused by *Botryosphaeria dothidea*, *Diaporthe* sp., *Pestalotiopsis microspora*, and *Alternaria alternata* resulted in substantial losses in the postharvest market [2,3,4]. *B. dothidea* and *Diaporthe* sp. were considered the most prevalent phytopathogens [3,5,6,7]. Pre-harvest spraying of chemical fungicides, such as Tebuconazole and Carbendazim, is the most cost-effective and practical method of prevention for kiwifruit rots [8]. Due to scientific concerns regarding human health and the ecological environment, as well as the development of resistant pathogens, substantial investigations need to be conducted on alternative management of postharvest soft rot.

Biological control, based on microbial antagonists to defend against pathogens, has been considered a sustainable and promising strategy [9,10]. Antagonistic microorganisms are safe and environmentally friendly, have a broad biocontrol spectrum and high efficacy towards diverse pathogens, do not result in resistant pathogen biotypes, and can be applied in conjunction with other measures [11,12]. Antagonist yeast is especially encouraged due to its unique characteristics: it is resistant to extreme conditions, generally has no pathogenic effects on the host, requires simple nutrition, and proliferates rapidly. Hence, antagonistic yeasts have been reported in a variety of fruits [13]. Yeast biocontrol mechanisms mainly involve competition for nutrition and space, direct parasitism, volatile antimicrobial compounds, and induction of host resistance [13,14,15]. Screening and investigation of the mechanisms of antagonistic yeast are fundamental for commercial usage. For kiwifruit, several studies had reported biocontrol yeast against postharvest diseases. *Meyerozyma caribbica* reduced blue mold in kiwifruit caused by *Penicillium expansum* [16], *Candida oleophila* [17], *Hanseniaspora uvarum* [18], and *Debaryomyces hansenii* [19] effectively inhibited gray mold caused by *Botrytis cinerea* and black rot caused by *A. alternata*. The biocontrol mechanisms of the above yeasts mainly relied on competition with pathogens for nutrition and space, and the induction-resistance response of the host. With *Meyerozyma guilliermondii*, some isolates were reported to control postharvest diseases, including ring rot, blue mold and gray mold of apples [20,21,22], blue mold of pears and mandarin fruit [23,24], fruit rot of strawberries [25], black spot of broccoli [26]. However, there were few reports on the antagonistic yeasts for kiwifruit soft rot, and whether *M. guilliermondii* could effectively control soft rot in kiwifruit required more screening and exploring of the biocontrol mechanism.

The objectives of this study were as follows: (1) to screen and identify an antagonistic yeast to control the soft rot of kiwifruit caused by *B. dothidea* and *D. actinidiae*; (2) to determine the inhibition effects of selected yeast treatments on pathogen spore germination and decay incidence of artificially infected kiwifruit; (3) to observe the adhesion to the pathogens and population dynamics in kiwifruit; (4) to detect potential biocontrol mechanisms including enzyme activity and expression level of corresponding genes in kiwifruit; and (5) to assess the yeast’s impact on natural decay and fruit quality of the stored kiwifruit.

## 2. Materials and Methods

### 2.1. Yeasts, Pathogens and Fruit

The 1113 yeast strains were kept in the Key Laboratory of Horticultural Plant Biology of Ministry of Education, Huazhong Agricultural University. Yeasts were transferred into 50 mL of peptone dextrose broth (PDB, extract of potatoes 200 g and dextrose 20 g per liter water) and incubated on a rotary shaker at 180 r/min at 25 °C for 20 h. Then, the yeast cells were centrifugated at 8000× *g* for 10 min at room temperature (RT), rinsed twice with sterile distilled water (SDW), and resuspended in SDW to the required concentration by a hemocytometer.

The fungal pathogens *B. dothidea* and *D. actinidiae* were isolated and purified from soft rot kiwifruit. Pathogens were cultivated on a potato dextrose agar medium (PDA, extract of potatoes 200 g, dextrose 20 g, agar 15 g per liter water) at 25 °C for 4 weeks. The spore suspension was prepared by washing the surface of mycelium with SDW, centrifugation, and resuspending spores to appropriate concentrations using a hemocytometer.

Yellow flesh cultivar (*Actinidia eriantha* × *A. chinensis* cv. Jinyan) were harvested at commercial maturity (soluble solids content, SSC, 7.5–8.0 °Brix) from the scientific research base of the Engineering Laboratory for Kiwifruit Industrial Technology (114° E, 30° N), Chinese Academy of Sciences (CAS). Healthy and uniform fruits were selected, superficially disinfected with 75% medicinal alcohol for 2 min, and then air-dried in plastic containers.

### 2.2. Screening of Antagonistic Yeast In Vitro and In Vivo

Mycelial discs (5 mm diameter) of actively growing *B. dothidea* and *D. actinidiae* were placed in the center of the PDA plate (90 mm), respectively, and each of 1113 yeast strains was inoculated 2 cm away from the pathogen disc. Plates containing only the pathogen served as control. Three replicates were conducted per yeast strain, and all cultures were incubated for 5 d at 25 °C. Yeast strains that restrict radial mycelium growth compared with control were selected.

According to the results of in vitro inhibition test, the selected antagonistic yeasts were subjected to in vivo biocontrol assay [27]. There were 16 and 121 strains selected for *B. dothidea* and *D. actinidiae*, respectively. Four wounds (approximately 2 mm deep and 5 mm wide) were performed using sterile toothpicks on the equatorial area of each fruit. Each wound was inoculated with 10 μL of each selected yeast suspension (1.0 × 10^8^ cells mL^−1^). SDW served as a control. After 2 h, 10 μL spore suspension of *B. dothidea* (1.0 × 10^5^ spores mL^−1^) and *D. actinidiae* (1.0 × 10^6^ spores mL^−1^) were added to each wound, respectively. Fruits were then placed into plastic boxes lined with wet paper towels and incubated at 25 °C and 90% relative humidity (RH). Decay incidences and lesion diameters were recorded and determined by the crisscross method at 5 days post-inoculation (dpi). For the final three candidate yeast strains for each pathogen, 20 wounds were used for one replicate, and each treatment was composed of three replicates and repeated twice. The decay incidence (%) was calculated as below: the number of decay wounds/total wounds per replicate × 100.

### 2.3. Antagonistic Yeast Identification

The yeast strain 37 that exhibited the best biocontrol activity against both two pathogens in vivo was identified. DNA was extracted using a Yeast Genomic DNA Extraction Kit (Solarbio, Beijing, China) according to the protocols. Identification was confirmed by DNA sequencing of the internal transcribed spacer (ITS) region and the D1/D2 domain of the 26S rDNA gene, the universal primers were as follows: ITS1 (5′-TCCGTAGGTGAACCTGCGG-3′) and ITS4 (5′-TCCTCCGCTTATTGATATGC-3′) [28]; NL1 (5′-GCATATCAATAAGCGGAGGAAAAG-3′) and NL4 (5′-GGTCCGTGTTTCAAGACGG-3′) [29]. PCR amplifications were conducted with the following conditions: 94 °C for 3 min; 30 cycles of 94 °C for 30 s, 55 °C for 30 s, 72 °C for 1 min; 72 °C for 5 min. The PCR products were sequenced by BGI Genomics (Beijing, China). Sequences were aligned with the NCBI BLAST searching tool (http://www.ncbi.nlm.nih.gov/BLAST (accessed on 9 September 2021)). A phylogenetic tree based on ITS sequences was constructed with the neighbor-joining algorithm in MEGA 11.

### 2.4. Detection of the Adhesion of Yeast to Pathogens

The adhesion of yeast strain 37 to two pathogens was observed according to a previous report [27] with modifications. 2 μL of spore suspension (1.0 × 10^5^ spores mL^−1^) of *B. dothidea* and *D. actinidiae* were inoculated at the center of PDA plates respectively. Simultaneously, microscope slides with PDA medium on the surface were inserted obliquely into the PDA plates, 1.5 cm away from the inoculation site. The plates were cultured at 25 °C for 3 d. When the mycelium initially expanded above the microscope slides, the yeast cell suspension (20 μL, 1.0 × 10^8^ cells mL^−1^) was dropped onto the mycelium and co-cultivated for 2 d. The mycelium above the slides was washed with SDW for 30 s after 24 h and 48 h, and directly observed under an optical microscope (Olympus BX51, Japan). Each treatment contained three replicates with four slides per replicate, and the observations were conducted twice.

### 2.5. Effect of Strain 37 on Spore Germination and Germ Tube Elongation of Pathogen

The effect of yeast strain 37 on spore germination was observed following Wang et al. [27] with modification. The yeast was inoculated into Erlenmeyer flasks containing 50 mL PDB, incubated in a rotary shaker for 48 h (180 r/min, 25 °C), and then prepared in three different treatments: strain 37 in PDB (1.0 × 10^8^ cells mL^−1^); strain 37 in SDW (1.0 × 10^8^ cells mL^−1^, washed twice with SDW); cell-free supernatant (CFS) filtered through a Millex-GP filter (0.22 μm, 33 mm, PES membrane). 100 μL of *B. dothidea* spore suspension (1.0 × 10^6^ spores mL^−1^) were inoculated into 800 μL PDB in a centrifuge tube, simultaneously 100 μL of the above three yeast treatments were added respectively, with SDW serving as a control. Then the tubes were transferred to a rotary shaker for 2 h (180 r/min, 25 °C). Spore germination and germ tube elongation were detected and measured with a microscope (BX51, Olympus, Japan). Each treatment consisted of three replicates (100 spores per replicate), and the test was repeated twice.

### 2.6. In Vivo Effect of Strain 37 on Soft Rot in Kiwifruit

The yeast strain 37 was prepared in three different treatments as described in Section 2.5, with SDW serving as a control. In vivo biocontrol essay was conducted the same as in Section 2.2. Each treatment was composed of three replicates (20 wounds per replicate) and performed twice.

### 2.7. Population Dynamics Essay of Strain 37 in Kiwifruit Wounds

The population dynamics essay of strain 37 in kiwifruit wounds was performed according to Zhang et al. [30]. Fruits were prepared (4 wounds per fruit) as described in Section 2.2. Each wound was pipetted with 10 μL strain 37 suspension (1.0 × 10^8^ cells mL^−1^). Kiwifruits were kept in plastic containers coated with wet paper towels (25 °C and 4 °C, 90% RH), and the population dynamics were assessed daily at 25 °C and every 3 d at 4 °C. Data from 2 h post-treatment served as 0 dpi. Wounded tissues were extracted with a sterilized punch (1 cm diameter) and ground with 10 mL SDW in a sterilized mortar. 10 μL of serial 10-fold suspensions were spread on the NYDA plate (beef extract 8 g; yeast extract 5 g; dextrose 10 g; agar 20 g per liter) and cultured at 25 °C for 2 d. Colonies were counted and population dynamics of strain 37 were expressed as Log_10_ CFU wound^−1^. The essay was performed with three replicates (8 wounds, 2 fruits for each replicate) and carried out twice.

### 2.8. Determination of Enzyme Activity and Antioxidant Content of Kiwifruit

Enzyme activity and antioxidant content of kiwifruit were determined according to Huang et al. [21] with little modification. Kiwifruits were treated sequentially with strain 37 (SDW as control) and *B. dothidea*, as described in Section 2.2. Healthy tissues surrounding the wounds (within 1.5 cm) were taken and mixed every day until 5 dpi. Samples were frozen immediately in liquid nitrogen and kept in the ultra-cold freezer at −80 °C. Each treatment contained three replicates and each replicate consisted of 6 kiwifruit. Defense enzymes activity of superoxide dismutase (SOD), catalase (CAT), phenylalanine ammonia-lyase (PAL), glutathione (GSH), and total phenol content were determined using corresponding test kits (Nanjing Jiancheng, China). Flavonoids content and cell wall degrading enzyme activity of β-galactosidase (β-Gal) and polygalacturonase (PG) were assessed by enzyme-linked immunosorbent assay (Elisa) kits (Feiya Biotechnology, Yancheng, China).

### 2.9. Fruit RNA Extraction and Gene Expression Analysis

The total RNA of corresponding kiwifruit samples (2.0 g) was extracted with the Hipure plant RNA mini kit (Magen, Guangzhou, China) following the protocols. The extracted RNA was determined by 1.0% agarose gel electrophoresis for purity and quantified with a spectrophotometer (DeNovix Inc., Wilmington, DE, USA). Template cDNA synthesis was performed with MonScript™ RTIII All-in-One Mix with dsDNase (Monad, Wuhan, China).

The transcript levels of target genes were conducted using MonAmp^TM^ ChemoHS qPCR Mix (Monad, Wuhan, China) following the instructions. A QuantStudio^TM^ Real-Time PCR System (Applied Biosystems, Waltham, MA, USA) was applied for the amplification with a PCR program: 95 °C for 10 min; 40 cycles of 95 °C for 10 s, 60 °C for 30 s. Melting curve analysis was performed at the end of the PCR to validate the amplification of single products. The primer sequences used for RT-qPCR analysis were shown in Table 1. The transcript level of target genes, *SOD*, *CAT*, *β-Gal*, and *PG*, was normalized using the 2 ^−ΔΔCT^ method with reference gene *Achn 107181* of kiwifruit [31,32]. The analysis was carried out with three biological and technical replicates.

### 2.10. Postharvest Biocontrol Evaluation

Due to the limitation of harvesting periods for different cultivars, *A. chinensis* cv. Jinmei was applied to evaluate the postharvest biocontrol efficiency of strain 37. Kiwifruits were obtained when SSC reached 7.5–8.0 °Brix in the research base of the Engineering Laboratory for Kiwifruit Industrial Technology, CAS. Fruits were randomly rinsed into strain 37 in PDB medium (1.0 × 10^7^ cells mL^−1^) for 1 min on the harvest day, untreated fruits served as a control. The fruits were then kept at 22 °C of RT and counted for natural decay incidence half a month after harvest, with each treatment containing three replicates and 30 fruits for each.

Another portion of the treated fruits was determined for soft-ripe quality when the firmness dropped to less than 1 kg of force (kgf). Firmness was tested by a texture analyzer (GS-15, Guss Manufacturing Pty Ltd., Cape Town, South Africa) after 1 mm of peeling at the equator [33]. A digital refractometer (PAL-1, Atago, Tokyo, Japan) was used to measure SSC and recorded as °Brix. The non-reducing sugar was hydrolyzed into reducing sugar by acid hydrolysis, and 3,5-dinitrosalicylic acid (DNS) for reducing sugar was then used to determine soluble sugar. Vitamin C content and titratable acidity were analyzed with 2,6-dichlorophenol-indophenol and sodium hydroxide titration, respectively. Each treatment was assessed with three replicates and 6 fruits for each.

### 2.11. Statistical Analysis

All statistical analysis was performed using SPSS Statistics 21 (SPSS Inc., Chicago, IL, USA), and the results were shown as mean ± standard error (SE). The significance of variances was calculated by independent samples *t*-test or one-way ANOVA with Duncan’s multiple range test (*p* < 0.05).

## 3. Results

### 3.1. Screening of Antagonistic Yeast

**Figure 1:**
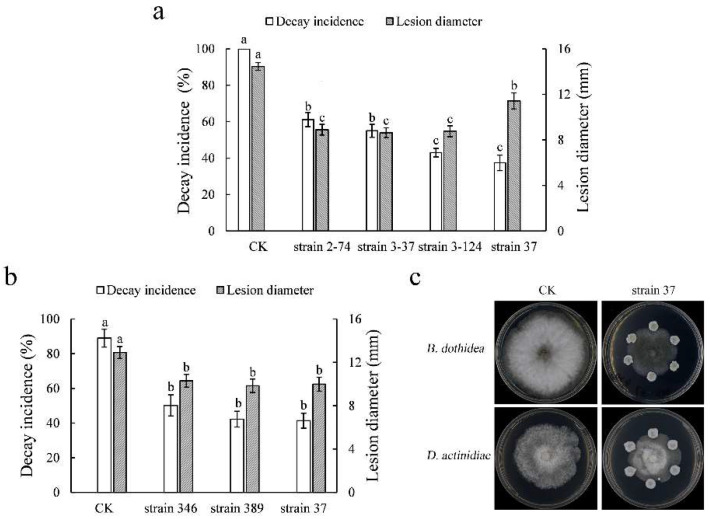
Inhibitory impact of selected candidate yeast strains on the soft rot of kiwifruit caused by *Botryosphaeria dothidea* (**a**) and *Diaporthe actinidiae* (**b**) in vivo and in vitro (**c**). The data is represented as mean ± standard error (SE). The letters above the columns show statistically significant differences (*p* < 0.05) as assessed with Duncan’s multiple range test.

There were 16 and 121 yeast strains selected for *B. dothidea* and *D. actinidiae* via in vitro antifungal test, respectively. Further in vivo tests screened out three candidate yeast strains for both pathogens, the inhibitory impact on the soft rot of kiwifruit was shown in Figure 1. Yeast strain 37 exhibited antifungal activity against both pathogens in vitro and in vivo, with the decay incidence caused by *B. dothidea* and *D. actinidiae* decreasing to 37.39% and 41.36%, respectively.

### 3.2. Identification of Antifungal Yeast Strain 37

**Figure 2:**
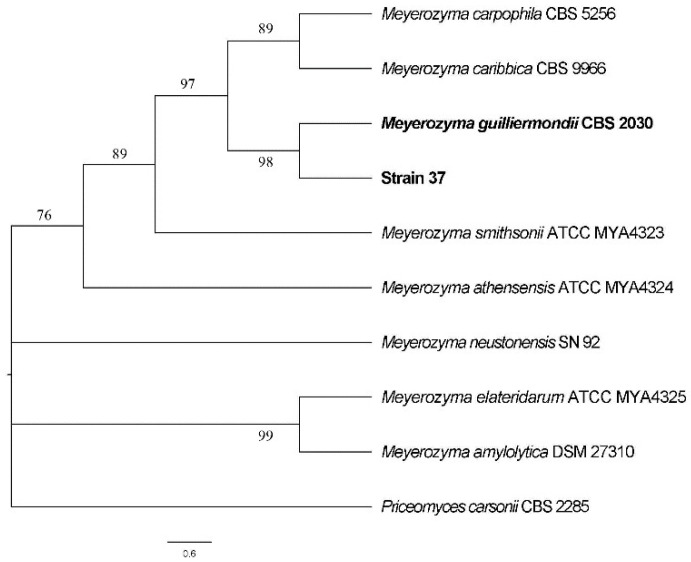
Phylogenetic tree of ITS sequence of strain 37.

According to the cluster analysis of the ITS sequence of strain 37 with several homologous strains from GenBank, strain 37 was in the same clade as *Meyerozyma guilliermondii* strain CBS 2030 (GenBank accession number: MH545918.1) in the phylogenetic tree (Figure 2). The 26S rDNA D1/D2 domain sequence analysis revealed that strain 37 had 100% identity with *M. guilliermondii* strain CBS 2030. These findings suggested that strain 37 is *M. guilliermondii*.

### 3.3. Adhesion of Yeast to Pathogens

**Figure 3:**
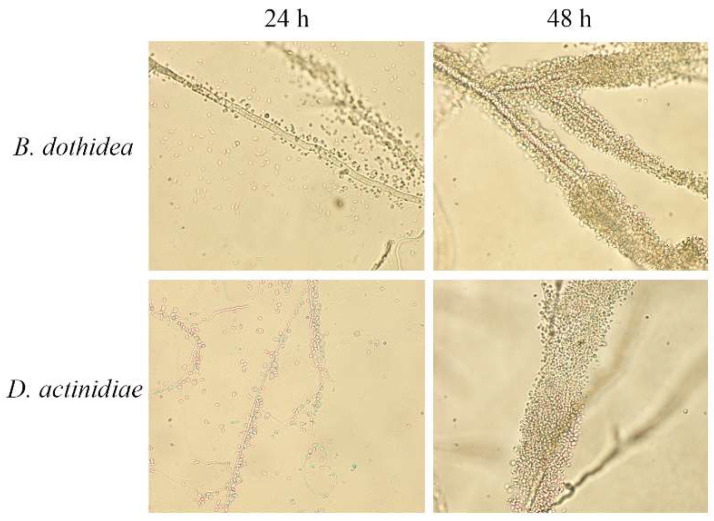
Adhesion of *Meyerozyma guilliermondii* 37 to *B. dothidea* and *D. actinidiae* (magnification: 400×) for 24 h/48 h after incubation at 25 °C.

After co-cultivation on PDA for 24 h and flushing with SDW, *M. guilliermondii* 37 adhered to the hyphae of *B. dothidea* and *D. actinidiae* closely. After 48 h, *M. guilliermondii* 37 adhered more densely to the hyphae (Figure 3).

### 3.4. Efficacy of M. guilliermondii 37 on Spore Germination of B. dothidea

**Figure 4:**
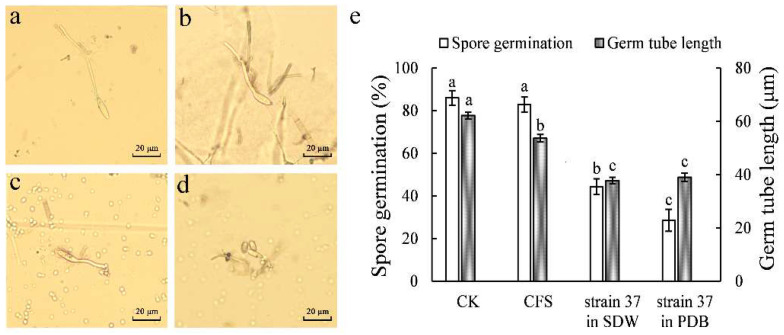
Efficacy of different treatments of *M. guilliermondii* 37 on the spore germination of *B. dothidea*. The spores were cultured in PDB medium CK (**a**); CFS (**b**); *M. guilliermondii* 37 suspensions in SDW (**c**); and *M. guilliermondii* 37 suspensions in PDB (**d**) for 2 h, respectively. Scale bar = 20 μm. Germination rate and germ tube length were assessed (**e**). The data are shown as mean ± SE. The letters above the columns show statistically significant differences (*p* < 0.05) as assessed with Duncan’s multiple range test.

The spore germination was significantly restrained by *M. guilliermondii* 37 suspensions (yeast in SDW/PDB) compared to control and CFS, the germination rate in *M. guilliermondii* 37 suspensions was 44.31% and 28.52%, respectively. The germ tube length of *B. dothidea* in *M. guilliermondii* 37 suspensions was also significantly lower than control and CFS (Figure 4).

### 3.5. Efficacy of M. guilliermondii 37 on Kiwifruit Soft Rot Caused by Two Pathogens

**Figure 5:**
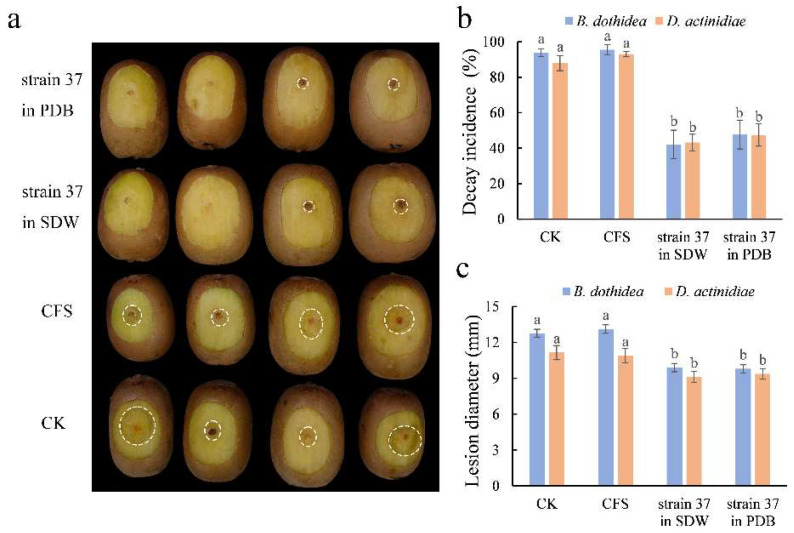
Effect of different treatments of *M. guilliermondii* 37 on kiwifruit soft rot caused by *B. dothidea* and *D. actinidiae*. Symptoms caused by *B. dothidea* (**a**); decay incidence (**b**); and lesion diameter (**c**) were evaluated after 5 days of treatment at 25 °C, RH 90%. The data is represented as mean ± SE. The letters above the columns show statistically significant differences (*p* < 0.05) as assessed with Duncan’s multiple range test.

When kiwifruit were pretreated with *M. guilliermondii* 37 suspensions (1.0 × 10^8^ spores mL^−1^) 2 h before the pathogen, both for *B. dothidea* and *D. actinidiae*, decay incidence (42.11~47.69%) was significantly lower than those pretreated with SDW (CK) and CFS (87.97~95.52%). The comparing result of the lesion diameter was consistent with the decay incidence (Figure 5).

### 3.6. Population Dynamics of M. guilliermondii 37 in Kiwifruit Wounds

**Figure 6:**
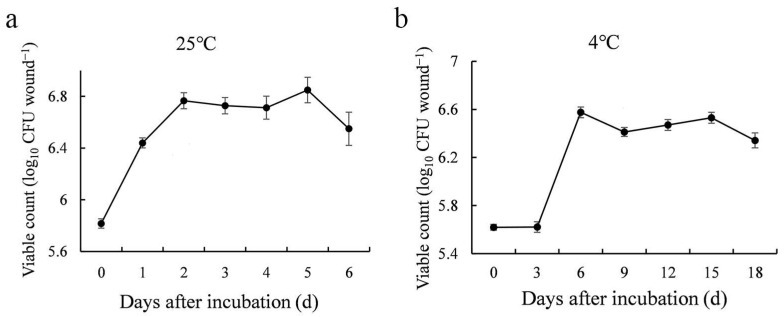
Population dynamics of *M. guilliermondii* 37 in the wounds of kiwifruit stored at 25 °C (**a**); and 4 °C (**b**). The vertical bars represent the SE of the mean.

Our result showed that *M. guilliermondii* 37 rapidly colonized kiwifruit wounds at both 25 °C and 4 °C (Figure 6). The population of *M. guilliermondii* 37 grew exponentially over the first 2 days at 25 °C (from 5.82 to 6.77 log_10_ CFU wound^−1^) and increased rapidly from 3 dpi to 6 dpi at 4 °C (from 5.62 to 6.58 log_10_ CFU wound^−1^). The population then remained relatively stable and declined slightly during the final period of the assay.

### 3.7. Impact of M. guilliermondii 37 on Enzyme Activity and Antioxidant Content of Kiwifruit

**Figure 7:**
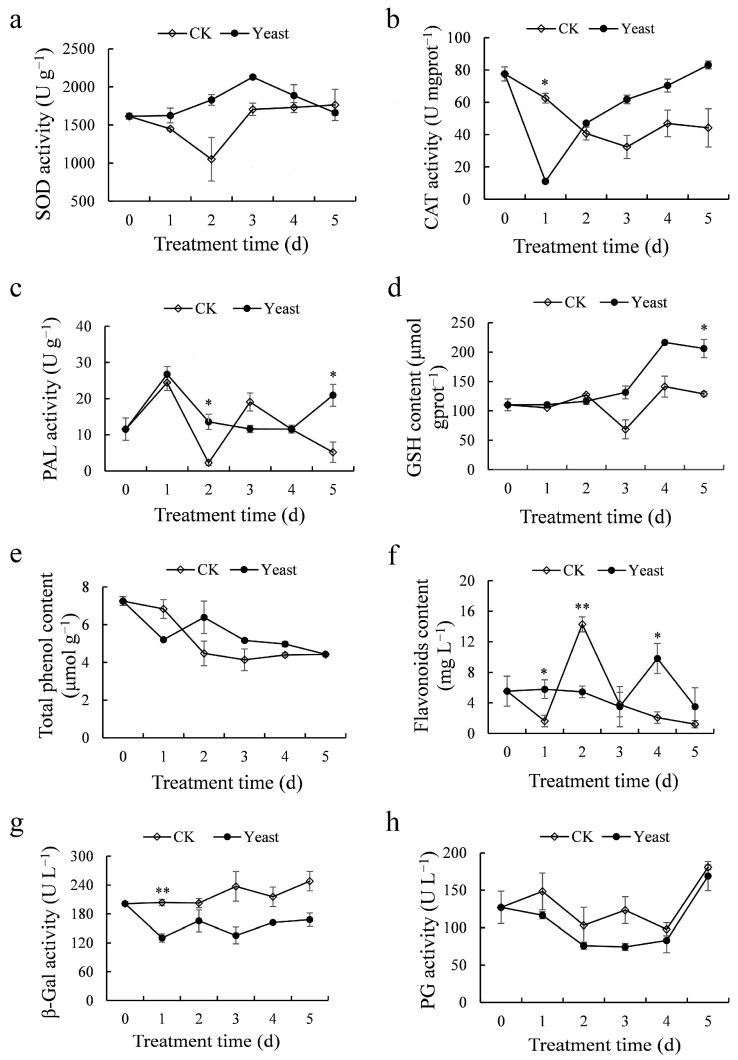
Effect of *M. guilliermondii* 37 on enzyme activity and antioxidant content in kiwifruit inoculated with *B. dothidea* for 5 days at 25 °C. (**a**–**h**) show changes in SOD, CAT, PAL, GSH, total phenol and flavonoids content, β-Gal, and PG, respectively. The data is represented as mean ± SE. * indicates the significant level of differences between treatments evaluated by Student’s *t*-test at *p* < 0.05, ** indicates the significance level of the differences at *p* < 0.01.

To confirm if *M. guilliermondii* 37 may promote kiwifruit resistance to the disease, the antioxidant enzymes (SOD, CAT, and PAL) activity, antioxidants (GSH, total phenol, and flavonoids) content, and cell wall-degrading enzymes (β-Gal and PG) activity were assessed. Compared to untreated fruits, the activity of SOD and CAT in *M. guilliermondii* 37 pretreated kiwifruit was higher for consecutive 4 days (Figure 7a,b); PAL activity was significantly (*p* < 0.05) higher at 2 dpi and 5 dpi (Figure 7c); GSH and total phenol content was higher for consecutive 3 days (Figure 7d,e); flavonoids content was significantly (*p* < 0.05) higher at 1 dpi and 4 dpi (Figure 7f); β-Gal and PG activity was lower for consecutive tested 5 days (Figure 7g,h). These results suggested that *M. guilliermondii* 37 elevated antioxidant enzyme activity, promoted antioxidant content to some extent, and suppressed two cell wall degrading enzymes activity, which could aid in resistance enhancement.

### 3.8. Determination of Gene Expression of Kiwifruit

**Figure 8:**
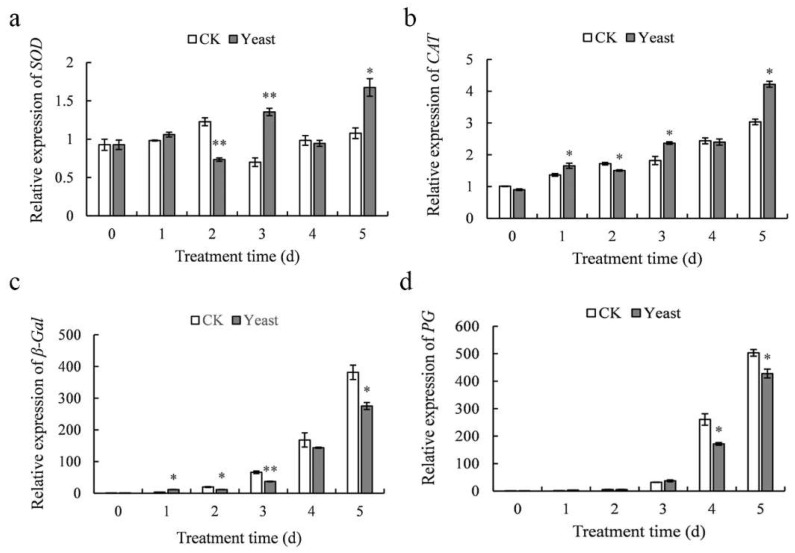
Effect of *M. guilliermondii* 37 on gene expression of *SOD* (**a**); *CAT* (**b**); *β-Gal* (**c**); and *PG* (**d**) in kiwifruit inoculated with *B. dothidea* for 5 days at 25 °C. The data are presented as mean ± SE. * denotes the significant level of differences between treatments according to Student’s *t*-test at *p* < 0.05, ** denotes the significant level of differences at *p* < 0.01.

The relative expression levels of *SOD*, *CAT*, *β-Gal*, and *PG* in the same samples were shown in Figure 8. Compared to the control, the relative expression of *SOD* in *M. guilliermondii* 37 pretreated fruits was significantly higher at 3 dpi and 5 dpi (Figure 8a); relative expression of *CAT* was significantly higher at 1, 3, and 5 dpi (Figure 8b). The relative expression of *β-Gal* and *PG* in control fruit increased continuously and achieved a peak transcriptional level at 5 dpi. In comparison, the relative expression of *β-Gal* in yeast-treated fruits was down-regulated from 2 dpi to 5 dpi (Figure 8c), and relative expression of *PG* was significantly (*p* < 0.05) down-regulated at 4 dpi and 5 dpi (Figure 8d). These findings were mainly aligned with the activity of the respective enzymes.

### 3.9. Postharvest Biocontrol Assay

To determine the biocontrol efficacy of *M. guilliermondii* 37 on postharvest soft rot, ‘Jinmei’ kiwifruit were soaked in yeast on the harvest day. The natural decay incidence of the yeast group (35.69%) was significantly (*p* < 0.05) lower than the control (55.17%), and there was no significant difference between the two groups in all tested soft-ripe quality parameters (Table 2). The results implied that *M. guilliermondii* 37 could effectively suppress soft rot while also preserving the soft-ripe quality of postharvest kiwifruit.

## 4. Discussion

Postharvest kiwifruit is susceptible to various fungal pathogens, especially soft rot caused by prevalent strains of *B. dothidea* and *D. actinidiae*, resulting in massive industry losses. Due to scientific concerns about consumer health and the ecosystem, as well as rising constraints on the application of chemical fungicides [34], extensive research has been conducted on safe and effective biocontrol agents. Several studies have found biocontrol yeast to be efficient against blue mold, gray mold, and black rot of kiwifruit. However, there are few reports on biocontrol yeast against kiwifruit postharvest soft rot caused by *B. dothidea* and *D. actinidiae*, as well as potential mechanisms. This study firstly screened and identified an antagonistic yeast that could reduce kiwifruit soft rot incidence. *M. guilliermondii* 37 was selected from 1113 yeast strains, nutrients and space competition and induction of kiwifruit resistance are the primary modes of action against pathogens. In addition, it reduced postharvest natural decay while having no negative effect on soft-ripe quality. These results indicated that *M. guilliermondii* 37 could be applied as a biocontrol agent to prevent soft rot in kiwifruit.

In our study, *M. guilliermondii* 37 in SDW and PDB significantly inhibited the spore germination of the pathogen in vitro and decay incidence in artificially infected kiwifruit. In contrast, CFS of *M. guilliermondii* 37 had no obvious inhibitory effect on pathogens, neither in the PDB medium nor in kiwifruit, indicating that strain 37 cannot produce direct antifungal compounds against the two pathogens. This is consistent with our finding in Result 3.1, which showed that there was no inhibition zone between the mycelium and yeast in the PDA medium. Similarly, culture filtrates of *Sporidiobolus pararoseus* ZMY-1 did not inhibit *B. cinerea*-caused decay on strawberries [28], and cell-free metabolites of *Metschnikowia citriensis* FL01 had no inhibitory effect on the growth of *Geotrichum citri-aurantii*, which caused sour rot of postharvest citrus [27].

Competition for nutrients and space is a primary mode of action of antagonistic yeast against postharvest fungal pathogens [13]. We observed the tenacious attachment of *M. guilliermondii* 37 to the hyphae of two pathogens after thorough washing with SDW, indicating yeast competition for space with the pathogen, as suggested by Di Francesco et al. [35], and *M. guilliermondii* had not before been known to adhere to fungal mycelium. In addition, *M. guilliermondii* 37 colonized kiwifruit wounds rapidly at both 25 °C and 4 °C, with an increasing population and maintaining a high level during the test periods, indicating that *M. guilliermondii* 37 could compete for nutrition and space with fungal pathogens in kiwifruit, which could be a possible antagonist mechanism. Coincidentally, a similar growth situation of *M. guilliermondii* had been reported to proliferate rapidly in apples [21] and pears [24]. Other biocontrol yeasts like *Candida oleophila* [17], *C. pseudolambica* [36], and *Wickerhamomyces anomalus* [30] also proliferated quickly in kiwifruit, peach, and pear, respectively. Moreover, the interval time of 2 h between *M. guilliermondii* 37 pretreatment and pathogen inoculation contributed to the inhibitory efficacy. Strain 37 was present in sufficient amounts at the appropriate location and time, and was able to reduce limited nutrients and space for the pathogen, preventing the invasion of *B. dothidea* and *D. actinidiae*. Similar biocontrol pretreatment (2 h interval) of other antagonistic yeasts had previously been documented [16,28,36]. The above-mentioned factors all contributed to the competition by *M. guilliermondii* 37. Particularly, growing in the field would stimulate a variety of survival strategies, and competition for the physical niche might acquire importance under these conditions.

The antagonistic yeast was considerably more effective when pretreated before pathogen inoculation, this observation inspired the hypothesis that yeast might trigger resistance responses in fruit. Resistance induction has been considered another important mechanism of biocontrol yeasts for preventing postharvest diseases [13,15]. We found that *M. guilliermondii* 37 pretreated kiwifruit exhibited increased enzyme activity of SOD, CAT, and PAL to some extent. *M. guilliermondii* was also observed to promote the above three enzyme activities in pears [37] and apples [21,22]. Antioxidant enzymes SOD and CAT play a functional role in the detoxification of reactive oxygen species (ROS) generated by plants in response to pathogenic fungi invasion. PAL is a crucial enzyme involved in the phenylpropanoid pathway, which directly participates in the synthesis of metabolites such as stilbenes, isoflavans, and coumarins that function to defend against pathogen attack [38], and it is positively related to plant disease resistance. Additionally, kiwifruit pretreated with *M. guilliermondii* 37 also exhibited more GSH content than the control. *M. guilliermondii* had been similarly reported to raise the GSH content of postharvest broccoli [26], while not in fruits. GSH is one of the major metabolites of the non-enzymatic ROS scavenging system, recognized as an important intracellular defense against oxidative damage. It participates in some crucial plant processes such as cell differentiation, cell senescence and death, and pathogen resistance [39]. Moreover, our yeast treatment also raised the total phenol and flavonoids content of kiwifruit, as the similar situation in *M. guilliermondii* treated apples [20] and pears [37]. Total phenol and flavonoids are secondary metabolites produced by plants in response to biotic or abiotic stresses and are linked to disease resistance mechanisms. Total phenol can scavenge free radicals [40], and flavonoids inhibit microbial cellulases, xylanases, and pectinases, and serve as a physical barrier against pathogen infection [41]. Overall, *M. guilliermondii* 37 enhanced defense-related enzymes activity (SOD, CAT, and PAL), and stimulated the accumulation of antioxidant substances (GSH, total phenol, and flavonoids), all of which induced kiwifruit resistance and contributed to pathogen defense.

β-Gal and PG are the two main enzymes involved in pectin hydrolysis, and pectin is the primary component of the cell wall. Few reports have examined the effect of *M. guilliermondii* and other biocontrol yeasts on the cell wall degradation enzymes of postharvest fruits. In our study, kiwifruit tissues were pretreated with *M. guilliermondii* 37 for 2 h before the pathogen, β-Gal and PG activity were suppressed compared to the control, and the gene expression of *β-Gal* and *PG* were significantly down-regulated, indicating that *M. guilliermondii* 37 inhibited cell wall degradation and fruit softening. This effect combined with increased antioxidant enzyme activity and antioxidant substance accumulation, contributed to elevating kiwifruit resistance to pathogens. The associated molecular mechanisms require additional investigation through transcriptomic analysis.

Few studies have been conducted on the postharvest soaking test of kiwifruit with biocontrol yeast to evaluate the control effect of yeast. In our study, the significantly decreased incidence of natural decay in the *M. guilliermondii* 37 group compared to the control suggested that *M. guilliermondii* 37 might be useful in kiwifruit postharvest management. Additionally, there were no differences in soft-ripe quality between treatments. A similar result was reported that *S. pararoseus* ZMY-1 immersion treatment reduced the natural decay of postharvest strawberries without affecting fruit quality [28]. Future investigations can be conducted to explore physical or chemical elicitors combined with *M. guilliermondii* 37 to enhance the biocontrol effect and reduce the need for chemical fungicides. Moreover, since the infection of soft rot often develops before harvest, effective measures may be implemented during the flower budding stage [4]. Thus, it is needed to explore more suitable timing and application methods.

## 5. Conclusions

In summary, this study firstly screened and identified an antagonistic yeast, *M. guilliermondii* 37, that effectively inhibited kiwifruit soft rot caused by *B. dothidea* and *D. actinidiae*, and significantly reduced natural decay in stored kiwifruit without affecting soft-ripe quality. It inhibited the pathogen spore germination rate significantly, attached tenaciously to pathogen mycelium, and colonized rapidly in kiwifruit. The biocontrol mechanism did not depend on antifungal compound secretion, depending on the competition with pathogens for nutrients and space, as well as kiwifruit resistance induction. These findings indicated the potential application of *M. guilliermondii* 37 as a biocontrol agent against soft rot in kiwifruit.

## Figures and Tables

**Table 1:** Sequences of primers used in RT-qPCR of relative gene expression in kiwifruit.

Gene	Primer Sequence 5’ → 3’
*SOD*	F: GAATGCTGAAGGTGCTGCTGTA
	R: TGGATCCTGATTTGCAGTTGTC
*CAT*	F: TTGCCCCTGCAACCTGTT
	R: CGATAATGGCAGGGCAGAAG
*β-Gal*	F: CACAGAAGACGGATCGAGTAAAG R: GGGTGCGTCAAATGTAGTCTTA
*PG*	F: CCAACGGCACTCAGATTCTAT R: TGTATTCGGACTGTCACCG
*Actin*	F: TGAGAGATTCCGTTGCCCAGAAGT R: TTCCTTACTCATGCGGTCTGCGAT

**Table 2:** Treatments on soft-ripe quality of kiwifruit.

Treatments	Decay Incidence (%)	Firmness (kgf)	SSC (°Brix)	Vitamin C (mg g^−1^)	Soluble Sugar (%)	Titratable Acidity (%)
Control	55.17 ± 1.99	0.65 ± 0.06	14.41 ± 0.37	0.67 ± 0.01	8.77 ± 0.14	1.60 ± 0.04
Yeast	35.69 ± 6.00 *	0.62 ± 0.06	14.86 ± 0.28	0.81 ± 0.05	8.65 ± 0.98	1.67 ± 0.04

Note: Data represent as mean ± SE. * indicates a significant level of difference between treatments as evaluated by the Student’s *t*-test at *p* < 0.05.

## Data Availability

Data is contained within the article.
